# Retrieving Land Surface Temperature from Hyperspectral Thermal Infrared Data Using a Multi-Channel Method

**DOI:** 10.3390/s16050687

**Published:** 2016-05-13

**Authors:** Xinke Zhong, Xing Huo, Chao Ren, Jelila Labed, Zhao-Liang Li

**Affiliations:** 1 ICube, UdS, CNRS, 300 Bld Sebastien Brant, CS10413, Illkirch 67412, France; x.zhong@unistra.fr (X.Z.); labed@unistra.fr (J.L.); 2 School of Computer and Information, Hefei University of Technology, Hefei 230009, China; huoxing@hfut.edu.cn; 3 College of Geometics and Geoinformation, Guilin University of Technology, Guilin 541004, China; renchao@glut.edu.cn; 4 Key Laboratory of Agri-Informatics, Ministry of Agriculture/Institute of Agricultural Resources and Regional Planning, Chinese Academy of Agricultural Sciences, Beijing 100081, China

**Keywords:** land surface temperature, hyperspectral thermal infrared, multi-channel method, land surface emissivity

## Abstract

Land Surface Temperature (LST) is a key parameter in climate systems. The methods for retrieving LST from hyperspectral thermal infrared data either require accurate atmospheric profile data or require thousands of continuous channels. We aim to retrieve LST for natural land surfaces from hyperspectral thermal infrared data using an adapted multi-channel method taking Land Surface Emissivity (LSE) properly into consideration. In the adapted method, LST can be retrieved by a linear function of 36 brightness temperatures at Top of Atmosphere (TOA) using channels where LSE has high values. We evaluated the adapted method using simulation data at nadir and satellite data near nadir. The Root Mean Square Error (RMSE) of the LST retrieved from the simulation data is 0.90 K. Compared with an LST product from the Spinning Enhanced Visible and Infrared Imager (SEVIRI) on Meteosat, the error in the LST retrieved from the Infared Atmospheric Sounding Interferometer (IASI) is approximately 1.6 K. The adapted method can be used for the near-real-time production of an LST product and to provide the physical method to simultaneously retrieve atmospheric profiles, LST, and LSE with a first-guess LST value. The limitations of the adapted method are that it requires the minimum LSE in the spectral interval of 800–950 cm^−1^ larger than 0.95 and it has not been extended for off-nadir measurements.

## 1. Introduction

Land Surface Temperature (LST) is a key parameter in climate systems. LST is used for Earth surface energy budget studies [1], numerical weather/climate forecasting [2], the retrieval of climate variables [3], soil moisture/evapotranspiration estimations [4], and generation of time-consistent LST product [5,6]. For severe weather forecasting application, the near-real-time LST can provide important diagnostic information [7]. Thermal infrared remote sensing has become an effective method to measure LST on large spatial scales [8,9].

Various methods to retrieve LST from satellite-based multispectral thermal infrared data include the following: the single-channel method [10], the Split-Window (SW) method [11,12,13,14], the multi-channel method [1,2,3,4,5,6,7,8,9,10,11,12,13,14,15,16,17], the multi-angle method [18], the physical-based day/night operational method [19], the Temperature and Emissivity Separation (TES) method [20], the multi-temporal physical method [21], the Kalman filter physical method [22], and the Two-Step Retrieval Method (TSRM) [23,24]. The single channel method requires good knowledge of the Land Surface Emissivity (LSE) at the channel used and an accurate atmospheric profile. The SW method requires accurate atmospheric water vapor content and LSE for land applications [8]. The use of the multi-channel method is limited by the uncertainty in LSE for the two mid-infrared channels (3–6 μm) being larger than that of the channels centered between 10 μm and 12 μm [25]. The multi-angle method suffers from the phenomenon of LSE and LST angular dependence [26]. The physical-based day/night operational method suffers from problems of geometry mis-registration, variations in the viewing zenith angle, and inaccurate atmospheric corrections [27]. The TES method, the multi-temporal physical method, and the Kalman-filter physical method require good atmospheric corrections [21,22,28]. The requirement of adequate channels and the TRSM method’s complex nature make it difficult to apply. The expected accuracy of a LST product from thermal infrared sensors is less than 1 K [29], however, this has not yet been achieved.

The hyperspectral thermal infrared data from sensors, such as the Infrared Atmospheric Sounding Interferometer (IASI) [30] and the Cross-track Infrared Sounder (CrIS) [31], have thousands of channels and provide a wealth of information on the atmosphere and the land surface. This type of data provides a new opportunity for methodological development in retrieving LST from satellite data.

The methods for retrieving LST from space-borne hyperspectral thermal infrared data can be classified into two types: empirical methods [32,33,34,35,36,37,38] and physical based methods [39,40,41,42,43,44]. The latter either require atmospheric profiles or are difficult to apply due to their complex nature. The empirical methods, which include the Artificial Neural Network (ANN) method and the principal component regression method, are based on a linear/nonlinear empirical relation between principal component amplitudes of the brightness temperature spectrum at the Top of the Atmosphere (TOA) and LST [32,33,34,35,36,37]. The principal component regression method and the ANN method are fast enough for near real-time applications [39]. However, the empirical methods require thousands of channels, which are not available for measurements because they contain damaged data at certain channels. It is required to develop a flexible multi-channel method for retrieving LST from hyperspectral thermal infrared data with less channels. Previously, we developed a multi-channel method to retrieve surface temperature for high emissivity surfaces from IASI data using brightness temperatures at TOA at 10 channels [38]. However, the multi-channel method for high emissivity surfaces mentioned above requires the assumption of blackbody LSE. The objective of this paper is to adapt the multi-channel method for retrieving LST from hyperspectral thermal infrared data for natural land surfaces while properly taking LSE into consideration.

The adapted multi-channel method can be used for retrieving LST for natural land surface from hyperspectral thermal infrared data containing damaged data at certain channels. The adapted multi-channel method is also promising for near-real-time production of LST products from hyperspectral thermal infrared data.

This paper is organized as follows: Section 2 adapts the multi-channel method using simulation data with typical LSE data of natural land surfaces. The sensitivity of the adapted method to instrumental noise and LSE is shown in Section 3. The evaluation of the adapted method using simulation data and satellite data is shown in Section 4, and the last section lists the conclusions.

## 2. Methodology

### 2.1. Multi-Channel Method

According to the multi-channel method for retrieving LST from hyperspectral thermal infrared data for high emissivity surfaces [38], LST can be retrieved from:

(1) $$T_{s}=w_{0}+\sum_{i=1:p}w_{i}Tb_{{}_{i}}^{}$$

where *T_s_* is the LST, *w_i_* (*i* = [1, p]) are the regression coefficients, and *Tb_i_* is the brightness temperature at TOA at channel *i*. The number of channels is p, and the center wavenumbers at channel *i* (*i* = [1, p]) and the *w_i_* (*i* = [0, p]) coefficients can be determined using simulation data with LSE of unity. The determined central wavenumbers of the channels are in the spectral interval of 800–1200 cm^−1^. The specifics for determining the w_i_ coefficients and the central wavenumbers of the channels are shown in [38]. This multi-channel method relies on the assumption that LSE is equal to one and can not be directly used for applications on natural land surfaces.

### 2.2. Adaptation for Natural Land Surfaces

#### 2.2.1. Adapted Multi-Channel Method

The previous multi-channel method is limited to applications on high emissivity surfaces. To extend the previous multi-channel method, we developed the adapted multi-channel method for retrieving LST from hyperspectral thermal infrared data for natural land surfaces. In the adapted multi-channel method, with the assumption that the LSE has high values in the spectral interval of [ν_a_, ν_b_] cm^−1^, LST can be retrieved from:

(2) $$T_{s}=\alpha_{0}+\sum_{i=1:n}\left(\alpha_{s,i}Tb_{{}_{s,i}}^{}+\alpha_{w,i}Tb_{{}_{w,i}}^{}\right)$$

where *α*_0_, *α_s,i_* (*i* = [1:*n*]) and *α_w,i_* (*i* = [1:*n*]) are the regression coefficients (also called *α_i_* in this paper), and *Tb_s,i_* (*i* = [1:*n*]) and *Tb_w,i_* (*i* = [1:*n*]) are the brightness temperatures at TOA at a channel-pair centered in the spectral interval of [ν_a_, ν_b_]. A channel-pair includes two nearby channels where the absorption of water vapor is strong in one channel and is weak in another channel. The *α*_0_, *α_s,i_*_,,_ and *α_w,i_* (*i* = [1:*n*]) coefficients and the central wavenumbers of the channels are determined using simulation data as described below. As a trade-off between the loss of information and the number of required channels, we only used the brightness temperatures at the channel-pairs, which represent the main feature of the brightness temperature spectrum at TOA in the spectral interval of [ν_a_, ν_b_].

#### 2.2.2. Analysis of the Variation in LSE

To determine the spectral interval where LSE has high values, we used typical LSE data from the Advanced Spaceborne Thermal Emission Radiometer (ASTER) emissivity library to study the variation in LSE. The wavelength of the LSE data in the ASTER library is from 714 cm^−1^ to 25,000 cm^−1^. The materials in the LSE data in the ASTER library include rocks, minerals, soils, vegetation, water bodies, meteorites, and manmade materials. Because pure pixels of rocks, minerals, meteorites, and manmade materials are rare in hyperspectral thermal infrared data with a spatial resolution of 12 km, we did not use the LSE data for these four types of materials for this analysis. Specifically, the materials of the LSE data used include water, snow, ice, three vegetation types, and 41 soils. The absorption of atmospheric ozone is strong in the spectral interval of 985–1071 cm^−1^. To eliminate the effect of atmospheric ozone, the spectral intervals used for this analysis were 800–985 cm^−1^ and 1071–1200 cm^−1^. The criteria for determining the spectral interval of [ν_a_, ν_b_] in Equation (2) is that the mean values of the channel LSEs are larger than 0.95 and the standard deviations of the channel LSEs are not larger than 0.01.

**Figure 1:**
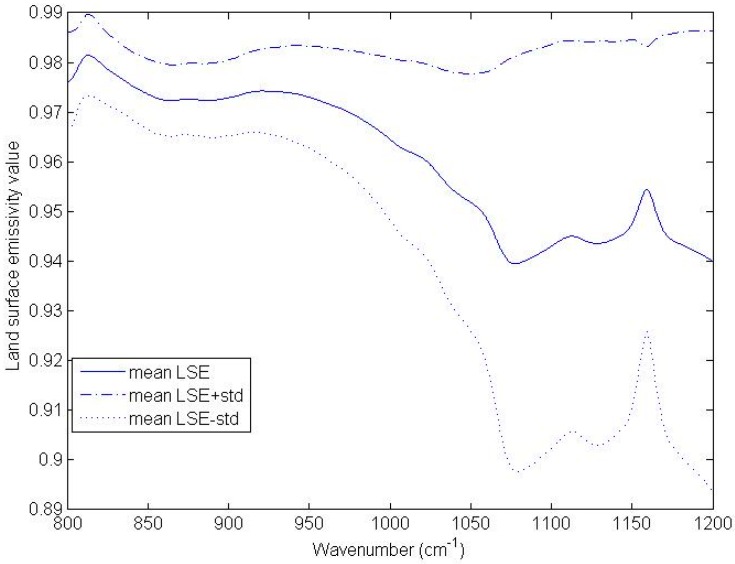
The mean values of the channel LSEs and the standard deviations of the channel LSEs as a function of wavenumber. (std = standard deviation of channel LSE).

The mean values of the channel LSEs and the standard deviations of the channel LSEs as a function of wavenumber are shown in Figure 1. The mean channel LSEs in the spectral interval of 800–950 cm^−1^ are larger than 0.95, and the corresponding standard deviations of the channel LSEs are approximately 0.01. The mean channel LSE decreases to about 0.943 in the spectral interval of 1071–1200 cm^−1^ and the standard deviation of the channel LSE increases to high values in the spectral interval of 1071–1200 cm^−1^, ranging between 0.03 and 0.045. We only considered the channels in the spectral interval of 800–950 cm^−1^ in the determination of the central wavenumbers of the channels.

#### 2.2.3. Determination of Central Wavenumbers of the Channels

To determine the central wavenumber of strong-absorption channels and weak-absorption channels in Equation (2), we simulated data using the Operational release for Automatized Atmospheric Absorption Atlas (4A/OP) [45,46] with the Middle-latitude-summer atmospheric profile extracted from the MODTRAN software. The atmospheric profile consists of atmospheric temperature and moisture profiles that have 35 layers from 1013 hPa to 0.01 hPa. The total precipitable water vapor and the bottom temperature of the atmospheric profile are 2.91 g/cm^2^ and 294.2 K, respectively. The LST and LSE for the simulation were 299.7 K and unity, respectively. 4A/OP was used to simulate the atmospheric radiative terms for measurements at nadir with the MODerate resolution atmospheric TRANsmission and radiance (MODTRAN) software build-in profiles. The spectral interval and spectral sampling frequency for the simulation were 800–950 cm^−1^ and 0.25 cm^−1^, respectively. The radiative transfer equation was used to simulate the brightness temperature spectrum at TOA for nadir observations with the atmospheric radiative terms from the 4A/OP model. With the brightness temperature data simulated using typical atmospheric profile data, we first selected the weak-absorption channels from each micro atmospheric window in the spectral interval of 800–950 cm^−1^. Then, we selected nearby strong-absorption channels, which had a brightness temperature difference larger than c K compared with themselves as the determined strong-absorption channels. The value of c for this simulated brightness temperature data is an empirical value of 2.5.

**Figure 2:**
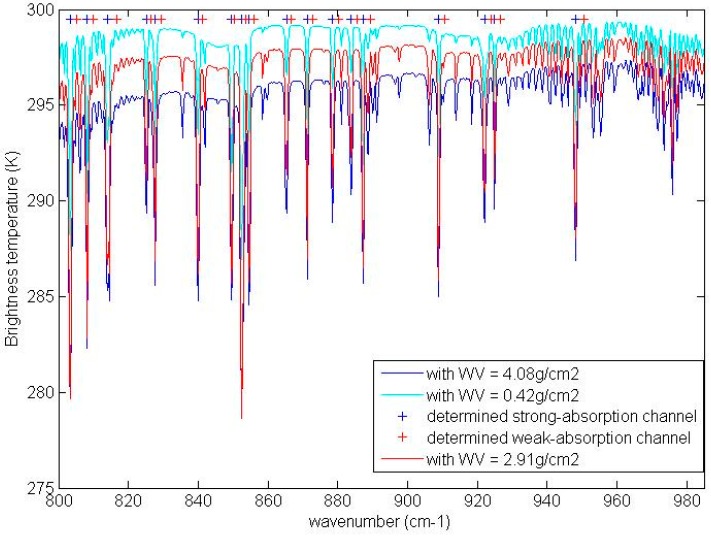
The central wavenumbers of the channels illustrated with the simulated brightness temperature spectrum at TOA (Top of Atmosphere). The bottom temperature is 294.2 K, and the LST (Land Surface Temperature) is 299.7 K.

The determined central wavenumbers of the channels and the simulated brightness temperature spectrum at TOA are shown in Figure 2. The 36 channels centered in the spectral interval of 800–950 cm^−1^ are used in this study.

To analyze the representativeness of the selected channels, we simulated brightness temperature data as above using the tropical and the sub-polar-winter atmospheric profile data extracted from MODTRAN. The total precipitable water vapors for the tropical profile and the sub-polar-winter profile are 4.08 g/cm^2^ and 0.42 g/cm^2^, respectively. The bottom temperatures for the tropical profile and the sub-polar-winter profile are 299.7 K and 257.2 K, respectively. This simulated brightness temperature data is shown in Figure 2. From Figure 2, we can see that the brightness temperatures at the selected channels are representative of the entire brightness temperature spectrum in 800–985 cm^−1^ for these typical atmospheric conditions_._

#### 2.2.4. Determination of the *α_i_* Coefficients

To determine the *α_i_* coefficients, we simulated a large amount of data using 4A/OP with typical atmospheric profiles from the Thermodynamic Initial Guess Retrieval (TIGR) database [47,48] as mentioned above. The atmospheric profile data and the LST data for the simulation to determine the *α_i_* coefficients were the same as those described in [38]. For each simulation condition, the LSE data for the simulation was the data referred to in Section 2.2.1. A random noise with a Noise Equivalent Temperature Difference (NE∆T) of 0.1 K was added to the simulated brightness temperature data at TOA. Using the large simulation data, we determined the *α_i_* coefficients in Equation (2) with the least square method.

**Figure 3:**
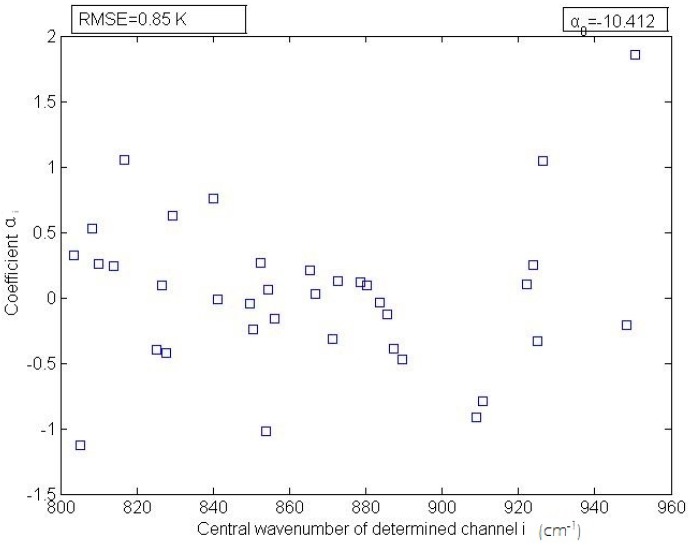
The *α_i_* coefficients and the central wavenumbers of the channels determined using the simulation data.

The determined *α_i_* coefficients are shown in Figure 3. The *α_i_* (*i* = [1, 36]) coefficients vary over a small range from −1.5 to 2.0.

## 3. Sensitivity Analysis

### 3.1. Sensitivity to Land Surface Emissivity

**Figure 4:**
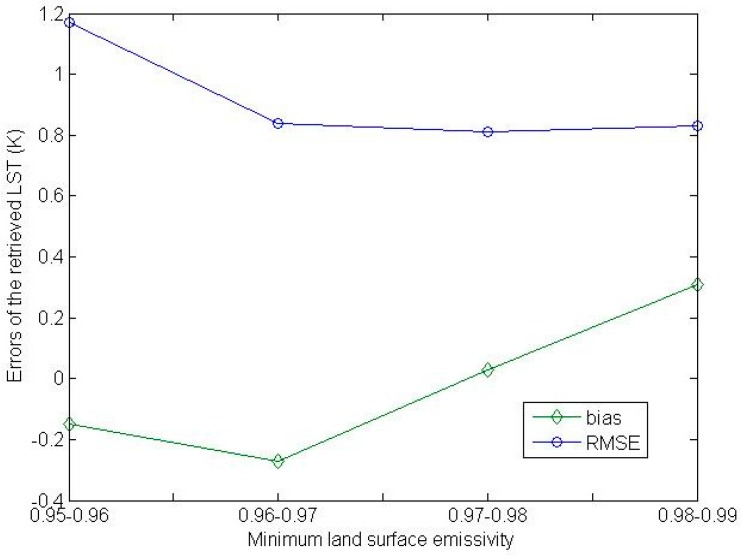
The errors of the retrieved LSTs for each simulation database as a function of the minimum LSE value in the spectral interval of 800–950 cm^−1^. (RMSE = Root Mean Square Error).

To analyze the sensitivity of the adapted method to LSE, we retrieved LSTs from independent simulation data using the adapted method and analyzed the variation of the error of the retrieved LSTs with the minimum LSE value in the spectral interval of 800–950 cm^−1^. The atmospheric profile data and the LST data for the independent simulation can be found in [38]. The total precipitable water vapor of the selected atmospheric profiles ranged from 0 g/cm^2^ to 5 g/cm^2^. The LSE data and the instrumental noise for the simulation are those mentioned in Section 2. To compute the statistics of the LST errors, we classified the simulation cases into four databases according to the minimum LSE value for which the minimum LSE values are shown in Table 1. The errors of the retrieved LSTs for each simulation database as a function of the minimum LSE value in the spectral interval of 800–950 cm^−1^ are shown in Figure 4.

As the minimum LSE value in the spectral interval of 800–950 cm^−1^ grows from approximately 0.95 to approximately 0.98, the bias of the retrieved LSTs for the simulation database with the corresponding LSE condition grows from −0.2 K to 0.3 K and the corresponding Root Mean Square Error (RMSE) of the retrieved LSTs decreases from 1.25 K to 0.85 K. The LST retrieved by the adapted multi-channel method has larger errors when the minimum LSE value is low. Note that the RMSE of the retrieved LSTs for the simulation data with a minimum LSE of 0.95 is less than 1.25 K.

The LST retrieved by the adapted multi-channel method is an increasing function of brightness temperatures at TOA at the selected channels and brightness temperature at TOA at a channel *i* is also an increasing function of LSE at this channel; as a result, the bias of the retrieved LST increases with the growing of the minimum channel LSE in the trend.

### 3.2. Sensitivity to Instrumental Noise

**Figure 5:**
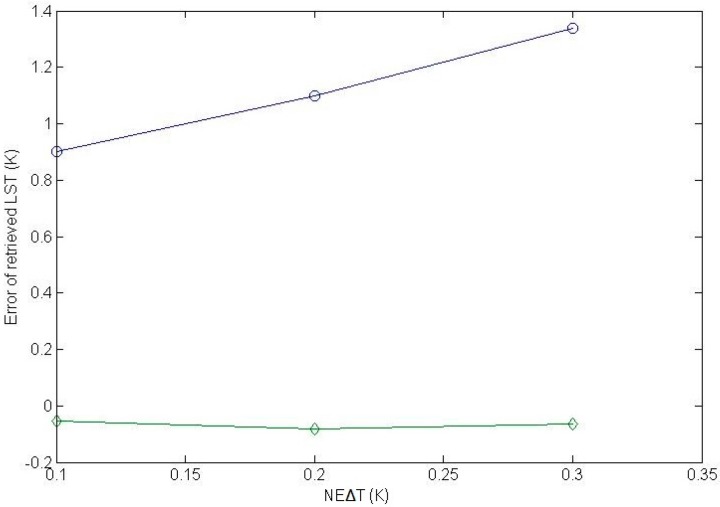
The errors of the LSTs retrieved from each noise-added simulation database as a function of the instrumental noise.

To conduct this sensitivity analysis, we created three simulation databases by adding noise to noiseless IASI data with NE∆T = 0.1 K, 0.2 K, and 0.3 K. The noiseless IASI data were created using the independent atmospheric profile data, the LST data, and the LSE data mentioned in Section 3.1. The method for adding noise to the noiseless simulation data can be found in [38]. The adapted multi-channel method was used to retrieve LST from the three simulation databases. Figure 5 depicts the errors of the LSTs retrieved from each simulation database as a function of the instrumental noise.

When the NE∆T for the simulation database was equal to that used to develop the adapted multi-channel method (0.1 K), the RMSE of the retrieved LSTs for the simulation database was 0.85 K. When the NE∆T for the simulation database was increased by 0.1 K and 0.2 K, the RMSEs of the retrieved LSTs for the corresponding simulation database increased by 0.35 K and 0.55 K, respectively. Therefore, the accuracy of the LST retrieved using Equation (2) is not significantly affected by the instrumental noise.

## 4. Evaluation

### 4.1. With Independent Simulation Data

We evaluated the accuracy of the adapted multi-channel method with the independent simulation data mentioned in Section 3.1. The central wavenumbers of the channels and the *α_i_* coefficients calculated in Section 2 were used to retrieve LST from the independent simulation data. The atmospheric profiles for the independent simulation were different from the atmospheric profiles mentioned in Section 2.

**Figure 6:**
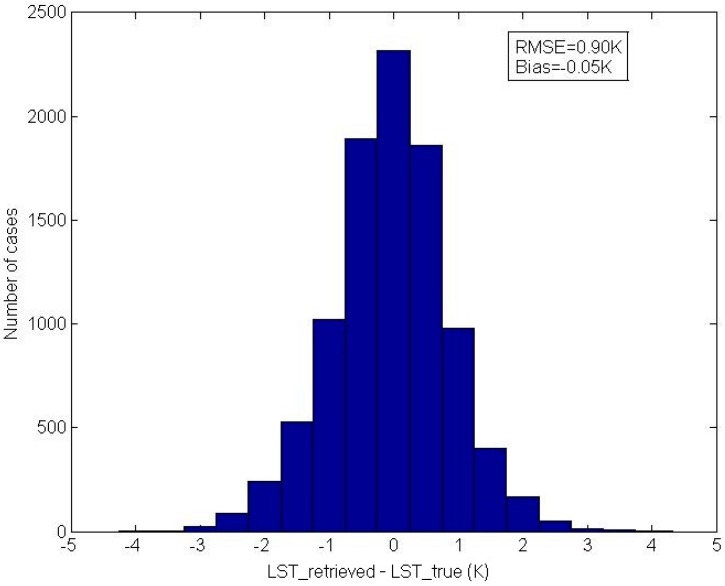
The error of the LST retrieved using Equation (2) from the independent simulation data (LST_retrieved = the retrieved LST and LST_true = the true LST).

The errors of the LSTs retrieved using Equation (2) for the independent simulation data are shown in Figure 6. The RMSE of the retrieved LSTs for the independent simulation data is 0.90 K. The error of the retrieved LST is consistent with the LST errors mentioned in other recent studies [21,35].

### 4.2. With Satellite Data

The simulation model itself has uncertainty; therefore, we evaluated its accuracy by comparing the LST retrieved by the adapted multi-channel method from Metop-A/IASI with the LST product from the Spinning Enhanced Visible and Infrared Imager (SEVIRI) on Meteosat.

**Figure 7:**
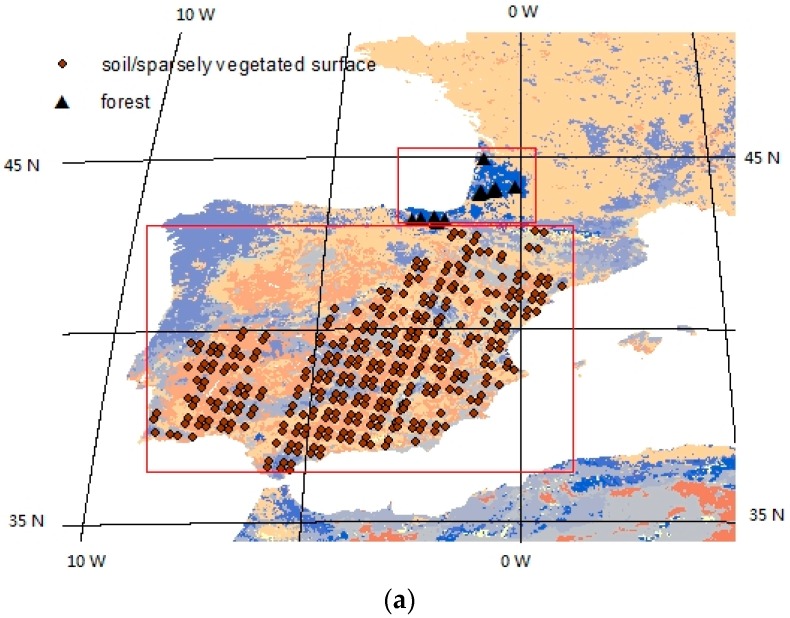

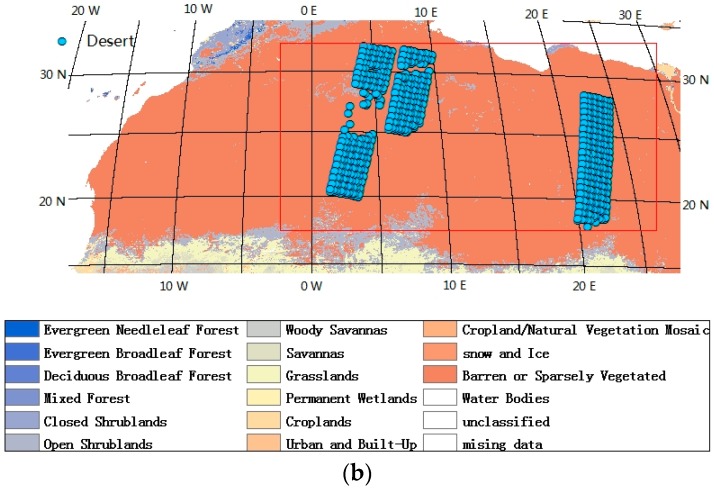
The target areas and target points in (**a**) Europe and in (**b**) North Africa used to evaluate the adapted multi-channel method with satellite data plotted on a land cover type map.

The target areas were the Sahara Desert, the Iberian Peninsula, and a forest area in the southwest of France and the north of Spain (Figure 7). The Sahara Desert has latitudes ranging from 7.0 W to 29.2 E and longitudes ranging from 17.2 N to 33.5 N. The desert surface is a homogeneous land surface. The Sahara Desert was selected because LST retrieved from hyperspectral thermal infrared data over desert surfaces have large uncertainties [28]. The Iberian Peninsula has longitudes ranging from 9.6 W to 0.6 E and latitudes ranging from 36.1 N to 44.0 N. The land surfaces in this area are mainly soil surfaces and sparsely vegetated land surfaces. This area was selected because (1) the sky is frequently clear over this area; and (2) various land surface types can be used for the evaluation. The targeted forest area has latitudes ranging from 42.7 N to 45.4 N and longitudes ranging from 3.4 W to 0.3 E.

The L1c data from Metop-A IASI has 8461 thermal infrared channels in the spectral interval of 645–2760 cm^−1^ with a spectral sampling frequency of 0.25 cm^−1^. The spatial resolution of an IASI image at the nadir point is 12 km. The scan angle at the end of each scan line is 48.98°. IASI on Metop-A scans the Mediterranean area in mid-morning orbits every day. Only IASI data with a viewing zenith angle less than 15° was used in this study.

SEVIRI on the Meteosat has eight infrared channels in the spectral interval of 769 cm^−1^ to 2564 cm^−1^. SEVIRI scans the hemispheric Earth surface every 15 min in geostationary orbit with a spatial resolution of 3 km. The SEVIRI/Meteosat LST product is used as a reference to evaluate the accuracy of the LST retrieved by the adapted multi-channel method from the IASI data. The SEVIRI/Meteosat LST product is retrieved using the generalized split-window method [49] with LSE as input data. The retrieval of LSE is based on the Vegetation Cover Method [50].

The five-minute Metop-A IASI L1c images on three clear days for each target area and their matched SEVIRI/Meteosat images were used for this evaluation. The sensing time of the selected IASI images for the Sahara Desert, for the forest areas, and for the Iberian Peninsula is shown in Table 2. The difference between the sensing time of an IASI image and that of the matched SEVIRI image was less than five minutes. IASI pixels with more than 95% clear-sky SEVIRI pixels were used for this evaluation. The criteria for spatially matching SEVIRI pixels and IASI pixels is that the distance between the center of IASI pixels and the center of SEVIRI pixels is less than 6 km. The matched points and the land cover type map are shown in Figure 7. The land cover types of the matched area in the Iberian Peninsula were primarily soil surfaces and sparsely vegetated surfaces. The land cover type of the targeted forest area was mainly evergreen needleleaf forest. In total, 1542 matched cases were used for the evaluation with the satellite data.

**Figure 8:**
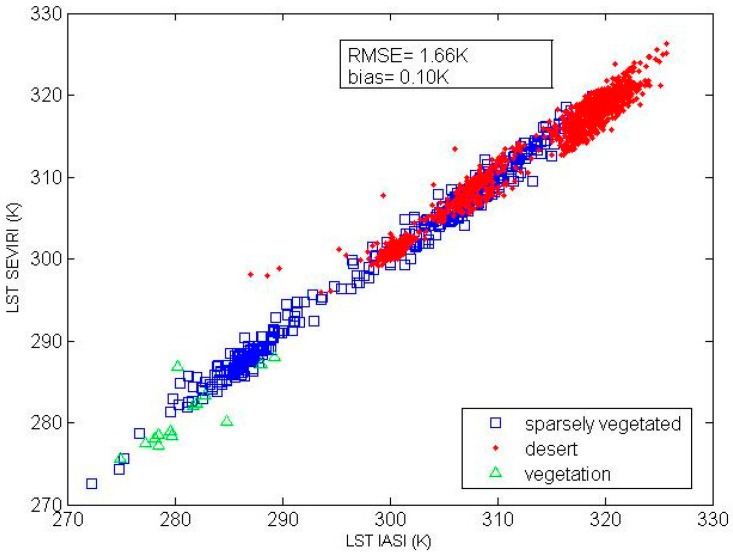
Comparison of the LST retrieved by the adapted multi-channel method from IASI/Metop-A data with the SEVIRI/Meteosat LST product on three clear days for each target area (SEVIRI = Spinning Enhanced Visible and Infrared Imager; LST IASI = the retrieved LST; and LST SEVIRI = the SEVIRI LST product).

The comparison of the LST retrieved by the adapted multi-channel method from the IASI data with the LST product from SEVIRI is shown in Figure 8. The root mean square difference between the LST retrieved from the IASI data and the LST product from SEVIRI is 1.66 K, and the mean difference between the two LST datasets is 0.1 K. On the whole, there is no large difference between the two LST datasets. Our finding is consistent with a recently reported finding on this LST difference [44]. 

**Figure 9:**
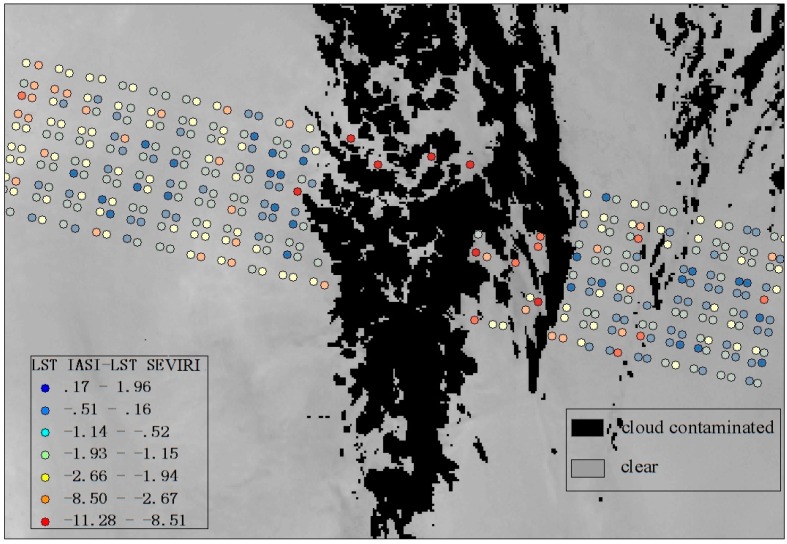
Difference between the LST retrieved by the adapted multi-channel method from IASI and the LST product from SEVIRI (IASI-SEVIRI) plotted on a quality map of the SEVIRI/Meteosat LST product over a typical part of the Sahara Desert on 2 November 2014 (LST IASI = the retrieved LST (K) and LST SEVIRI = the SEVIRI LST product (K)).

The spatial pattern of the difference between the LST retrieved from the IASI data by the adapted multi-channel method and the LST product from SEVIRI on 2 November 2014 over the Sahara Desert is shown in Figure 9. The larger LST differences are near the cloud-contaminated area, and the maximum LST difference is 11 K. As expected, the adapted multi-channel method cannot be applied to cloud-contaminated hyperspectral thermal infrared data. The large error of the LST retrieved from IASI for cloudy atmospheric conditions is also reported in a recent study [30].

## 5. Conclusions

Assuming the channel LSEs have large values in the spectral interval of 800–950 cm^−1^, we adapted the multi-channel method to retrieve LST from hyperspectral thermal data for natural land surfaces using 36 channels centered in the spectral interval of 800–950 cm^−1^ with simulation data. Then, we analyzed its sensitivity to LSE and instrumental noise using simulation data. Finally, we evaluated the accuracy of the adapted multi-channel method using simulation data at nadir and satellite data near nadir. This work draws the following conclusions:

(1) LST can be retrieved by the adapted multi-channel method from the simulation data with an RMSE of 0.90 K using hyperspectral thermal infrared data from only 36 channels.
(2) As the minimum LSE in the spectral interval of 800–950 cm^−1^ decreases from 0.98 to 0.95, the error of the LSTs retrieved by the adapted multi-channel method for the simulation data with the corresponding LSE condition increases from 0.85 K to 1.25 K. In addition, the impact of the instrumental noise is approximately three times its magnitude.
(3) The difference between the LST retrieved by the adapted multi-channel method from the IASI/Metop-A data and the LST product from the SEVIRI/Meteosat is approximately 1.6 K on average.

The adapted multi-channel method can be used for near-real-time retrieval of LST from hyperspectral thermal infrared data and to provide the physical method to simultaneously retrieve atmospheric profiles, LST, and LSE with first-guess LST value in the future. The limitations of the adapted multi-channel method are that it requires minimum channel LSE in the spectral interval of 800–950 cm^−1^ has value larger than 0.95 and it has not been extended for off-nadir measurements yet.

## Figures and Tables

**Table 1:** Minimum channel LSE values for the four databases for analysis of the sensitivity of the adapted method to LSE.

No. of Database	1	2	3	4
Minimum channel LSE values	[0.95, 0.96]	[0.96, 0.97]	[0.97, 0.98]	[0.98, 0.99]

**Table 2:** The sensing time of the selected IASI images for the three target areas.

Target Area	Sensing Time 1	Sensing Time 2	Sensing Time 3
the Sahara desert	2014-08-01 08:15	2014-05-03 09:15	2014-11-02 09:30
the forest areas	2015-04-05 21:00	2015-08-09 20:55	2015-10-25 21:02
the Iberian Peninsula	2014-05-05 10:10	2014-08-09 10:30	2014-11-10 10:00

## Abbreviations

The following abbreviations are used in this manuscript:

LST	Land Surface Temperature
LSE	Land Surface Emissivity
TES	Temperature and Emissivity Separation
TRSM	the Two Step Retrieval method
TOA	Top of Atmosphere
4A/OP	Operational release for Automatized Atmospheric Absorption Atlas
TIGR	Thermodynamic Initial Guess Retrieval
NE∆T	Noise Equivalent Temperature Difference
IASI	the Infrared Atmospheric Sounding Interferometer
CrIS	the Cross-track Infrared Sounder
SEVIRI	the Spinning Enhanced Visible and Infrared Imager
ANN	Artificial Neural Network
RMSE	the Root Mean Square Error
